# Antimicrobial resistance profile of *Staphylococcus aureus* isolated from patients with infection at Tikur Anbessa Specialized Hospital, Addis Ababa, Ethiopia

**DOI:** 10.1186/s40360-018-0210-9

**Published:** 2018-05-21

**Authors:** Sileshi Tadesse, Haile Alemayehu, Admasu Tenna, Getachew Tadesse, Tefaye Sisay Tessema, Workineh Shibeshi, Tadesse Eguale

**Affiliations:** 1 Yekatit 12 Hospital Medical College, P.O. Box157, Addis Ababa, Ethiopia; 2 Aklilu Lemma Institute of Pathobiology, Addis Ababa University, P.O. Box 1176, Addis Ababa, Ethiopia; 3 Department of Internal Medicine, School of Medicine, College of Health Sciences, Addis Ababa University, Churchill Avenue, P.O.Box 9086, Addis Ababa, Ethiopia; 4 Department of Biomedical Sciences, College of Veterinary medicine and Agriculture, Addis Ababa University, P.O. Box 34, Debrezeit, Ethiopia; 5 Institute of Biotechnology, College of natural and Computational sciences, Addis Ababa University, P.O. Box, 1176, Addis Ababa, Ethiopia; 6 Department of Pharmacology and Clinical Pharmacy, School of Pharmacy, College of Health Sciences, Addis Ababa University, Churchill Avenue, P.O. Box 9086, Addis Ababa, Ethiopia

**Keywords:** *Staphylococcus aureus*, Methicillin resistant *Staphylococcus aureus*, Surgical site infection, Drug resistance, Otitis media

## Abstract

**Background:**

*Staphylococcus aureus* is one of the major pathogens of public health importance responsible for various forms of infection. Development of resistance to commonly used antimicrobials limited treatment options against infections due to this pathogen. Antimicrobial resistance profile of *Staphylococcus aureus* isolated from patients with surgical site infection and ear infection and corresponding nasal swab was investigated in Tikur Anbessa Specialized Hospital (TASH), Addis Ababa, Ethiopia.

**Methods:**

Wound and corresponding nasal swabs from patients with surgical site infection from general surgery ward (*n* = 14), orthopedic ward (*n* = 21) and those with otitis media (*n* = 59) from Ear Nose and Throat (ENT) ward were cultured for *S. aureus* isolation according to standard procedures from December 2013 to June 2014. Isolates were investigated for susceptibility to panel of 17 antimicrobials using Kirby Bauer disc diffusion assay. Susceptibility to methicillin was phenotypically determined based on sensitivity of isolates to cefoxitin and oxacillin.

**Results:**

A total of 79 *S. aureus* isolates were recovered from 54(57.4%) of patients. The isolates were resistant to ampicillin (100%), oxacillin and cefoxitin (68.4%, each), clindamycin (63.3%), cephalothin (59.5%), tetracycline (57%), sulfamethoxazole + trimethoprim and bacitracin (53.2%, each), and erythromycin (51.9%). Resistance to two or more antimicrobials was recorded in 74 (95%) of the isolates, while resistance to 3 or more antimicrobials was detected in 65(82.3%) of the isolates. Fifty-four (68.4%) of the isolates were methicillin resistant *S. aureus* (MRSA). Rate of occurrence of MRSA was more common among isolates from surgical wards (*p* < 0.001) compared to those from ENT ward. High level of multi-drug resistance (MDR) was detected more commonly among isolates from orthopedic ward than those from general surgical ward and patients with ear infection (p < 0.001). One of the isolate cultured from wound swab of a patient with surgical site infection from orthopedic ward was resistant to all of the 17 antimicrobials tested.

**Conclusion:**

*S. aureus* isolates from patients in TASH exhibited resistance to majority of antimicrobials commonly employed for the treatment of staphylococcal infections which calls for urgent need of prudent use of antimicrobials and the need for implementation of effective infection control practices to hamper spread of MDR *S. aureus*.

## Background

*Staphylococcus aureus* is a normal flora associated with skin, skin glands and mucous membrane of almost all warm blooded animals and about 30% of the human population is colonized by *S. aureus* [1]. It is also a leading cause of life- threatening blood stream infection, osteoarticular, skin, soft tissue, respiratory tract, device- associated and surgical site infections particularly in immunocompromised, young and elderly patients [2]. The ability of *S. aureus* to invade the host immune system through various virulence factors and its rapid acquisition of multi-drug resistance phenotype, makes it one of the most notorious organism among gram positive bacterial pathogens [3].

The burden of infection with antimicrobial resistant strains of pathogens involves increased risk of mortality, increased hospital stay, and related attributable costs compared to infection with antimicrobial susceptible pathogens [4]. *S. aureus* is reported to be the most common cause of nosocomial infections and is particularly responsible for majority of surgical site infections [5]. In a previous study in northern Ethiopia, the rate of surgical site infection accounted for 10.2% and *S. aureus* was shown to be the leading bacterial pathogen responsible for surgical site infection [6]. Similarly, *S. aureus* was the most frequently isolated pathogen among patients with otitis media at Bahir Dar, northwest Ethiopia [7], Ayder teaching and Referral Hospital, Mekelle, northern Ethiopia [8] and isolates were resistant to several antimicrobials.

Multidrug resistant (MDR) strains of *S. aureus*, particularly methicillin resistant *Staphylococcus aureus* (MRSA) have potential of rapid spread in a given health facility through colonized or infected patients or health personnel as well as contaminated environments in the facilities, unless there is strict infection control strategy [9]. Recent study showed that infection with MDR strains of *S. aureus* is associated with prolonged length of hospital stay and increased mortality [10]. MRSA is one of the major public health threats globally. Antimicrobial resistance in MRSA is associated with acquisition of large mobile genetic element called staphylococcal cassette chromosome (SCCmec) which carries the central determinant for a broad spectrum beta-lactam resistance encoded by *mec*A or *mec*C genes [11, 12]. Studies showed widespread distribution of MRSA in various countries particularly in hospital environments [13–15] as well as occurrence of community and livestock associated MRSA [16–18]. In addition to *mec*A and *mec*C genes, SCC was reported to carry several other drug resistance genes which confer resistance to mercury, kanamycin, erythromycin, spectinomycin and fusidic acid [19]. Moreover, recent studies showed dramatic increase in the development of resistance to vancomycin, the other alternative drug for the treatment of infections caused by gram positive organisms [20].

It has been shown that overall epidemiology, pathophysiology and clinical manifestations of *S. aureus* vary significantly among different countries and different regions of the same country [2]. In most developing countries like Ethiopia, the potential public health threat due to antimicrobial resistance is high because of the fact that antimicrobial agents can easily be purchased without prescription [21], lack of coordinated routine surveillance of antimicrobial resistance, poor laboratory capacity, and poor infection control mechanisms by health facilities [22]; contributing to the emergence and spread of antimicrobial resistance [23]. Knowledge on the antimicrobial susceptibility status of circulating pathogens in a given health facility is important for better management of infectious pathogens particularly where routine culture and sensitivity testing is not practiced. Recent study at Yekatit 12 Hospital, Addis Ababa, showed isolation rate of *S. aureus* from 14.3% of clinical specimens, of which over 50% of the isolates were MDR and 17.5% were MRSA [24]. Scant information is available on the occurrence and antimicrobial susceptibility of *S. aureus* in patients with surgical site infection and those with otitis media in Ethiopia. Thus, the present study reports occurrence and antimicrobial resistance profile of *S. aureus* among patients with surgical site infection and ear infection and corresponding nasal swab at Tikur Anbessa Specialized Hospital (TASH), Addis Ababa, Ethiopia.

## Methods

### Study setting, study design and subjects

The study was conducted at Tikur Anbessa Specialized Hospital, a tertiary teaching hospital of Addis Ababa University, from December 2013 to June, 2014. The hospital provides diverse services for patients from different parts of the country. The study design was a hospital based cross-sectional study in which hospitalized patients from general surgical ward and orthopedic ward as well as outpatients from ear, nose, and throat (ENT) ward were involved. Patients with clinical evidence of surgical site infection having surgical wound with pus discharge, serous or seropurulent discharge, signs of sepsis and diagnosed for surgical site infection from general surgical and orthopedic wards were enrolled. These patients were hospitalized for various period and 25(71%) of them were treated with ceftriaxone and others were treated with antimicrobials such as metronidazole, vancomycin, ceftazidime, cloxacillin and gentamicin whereas there was no information on recent history of antimicrobial therapy for 5(14.3%) of the patients. In addition, patients with acute and chronic otitis media with clinically proven discharge from ENT outpatient ward were randomly recruited. Majority of these patients 34(61%) had history of recent treatment with ciprofloxacin and hydrogen peroxide, 4(6.8%) with amoxicillin+clavulanic acid while the rest 19(32.2%) had no history of recent antimicrobial therapy. Only patients who were willing to give their consent to participate in the study were involved.

### Sample collection and isolation of *S. aureus*

A total of paired swab samples from 94 patients; wound swab from those with surgical site infection (*n* = 14 from general surgical ward; *n* = 21 from orthopedic ward) and ear swab from patients with otitis media having clinical symptom of ear discharge (*n* = 59) and corresponding nasal swabs from all patients (*n* = 94) were collected. The wound site and ear were first cleaned with sterile saline to remove any purulent debris. Sterile cotton swab was moistened with normal saline and rotated three times on the wound surface and ear opening and placed in test tubes containing 10 ml of sterile Trypton Soya Broth(TSB), (BD, Diagnostic Systems, Heidelberg, Germany). Samples were transported in the ice box to the Microbiology Laboratory of Aklilu Lemma Institute of Pathobiology, Addis Ababa University within 3–4 h of collection and were immediately incubated at 37 °C overnight.

After overnight growth in TSB, loopful of the suspension was streaked into mannitol salt agar (Oxoid, Basingstoke, Hampshire, England). Then the plates were incubated at 37 °C for 24 h and bacterial colonies with typical characteristics of *S. aureus* (i.e. colonies with golden yellow pigmentation on mannitol salt agar) were subjected to subsequent biochemical tests involving Gram stain, catalase, and coagulase tests for confirmation. *Staphylococcus aureus* ATCC25923 was used as a reference strain.

### Antimicrobial susceptibility testing

Antimicrobial susceptibility test for *S. aureus* was carried out against a panel of 17 antimicrobials using Kirby Bauer disc diffusion method according to Clinical and Laboratory Standards Institute guidelines [25] on Mueller Hinton agar (MHA) (Oxoid, Basingstoke, England). The bacterial culture was grown in TSB for 4–5 h at 37 °C and the inoculum density was adjusted with 0.5 McFarland standard. A sterile cotton swab was dipped into the suspension and it was pressed against the sides of the tube to avoid excess inoculum. The inoculum was evenly spread on MHA plate and kept for 15 min before antimicrobial discs were dispensed. The plates were then incubated at 37 °C for 24 h and the diameter of zone of inhibition was measured using plastic transparent ruler. The interpretation of the categories of susceptible, intermediate or resistant was based on the CLSI guidelines [25]. Reference strain of *S. aureus* ATCC25923 was used as a quality control organism. The following antimicrobials with disk potencies (μg) were used (Sensi-Discs, Becton, Dickinson and Company, sparks, MD): oxacillin (Ox; 1), cefoxitin (Fox;30), cephalothin (Cf; 30), bacitracin (B;10 IU), clindamycin (Da; 2), ampicillin (Amp; 10 μg), amoxicillin-clavulanic acid (Amc; 30), ceftriaxone (Cro; 30), chloramphenicol (C) (30 μg), ciprofloxacin (Cip;5), erythromycin (E;15), gentamicin (Gm; 10), amikacin (An; 30), sulphamethoxazole-trimethoprim (Sxt;25), doxycycline (Do,30), tetracycline (Te; 30), and nitrofurantoin(Nitro;300).

### Data analysis

The chi-square test was employed to investigate association of sex and age of patients with carriage rate of *S. aureus* and MRSA. One way analysis of variance and student t-test were used to compare the difference in the level of multidrug resistance in *S. aureus* originating from various wards and specimens. The difference between the means was considered significant at *p* < 0.05.

## Results

### *Staphylococcus aureus* infection rate

Of all 188 specimens cultured for *S. aureus,* 79 (42.02%) were positive*. S. aureus* was detected from 54 (57.4%) of the 94 patients examined either from wound/ear discharge swab or nasal swab. Rate of recovery of *S. aureus* was higher among specimens obtained from patients with otitis media (44.1% from ear discharge and 50.9% from nasal swab) compared to wound and nasal swab of patients with surgical site infection which ranged from 28 to 38%. However, overall *S. aureus* carriage rate per patient was almost similar among patients from various wards (Table 1). There was no statistically significant difference in occurrence of *S. aureus* among different age group and sex (Table 2).

**Table 1:** Relative isolation rate of *S. aureus* from wound and nasal swab of patients with surgical site infection and ear discharge swab and nasal swab of patients with otitis media

Patient category	No. of patients	No. of patients positive from one or more specimen (%)	Specimen	No. examined	No. (%) positive	Total no. of isolates
General surgical ward	14	8(57.1)	Wound swab	14	4(28.6)	8
			Nasal swab	14	4(28.6)	
Orthopedic ward	21	10(47.6)	Wound swab	21	8(38.1)	15
			Nasal swab	21	7(33.3)	
ENT ward	59	36(61)	Ear discharge	59	26(44.1)	56
			Nasal swab	59	30(50.9)	
Total	94	54(57.5)		188	79(42.02)	79

**Table 2:** Sociodemographic characteristics of patients with surgical site infection, ear infection and carriage rate of *S. aureus* at Tikur Anbessa Specialized Hospital

Characteristics	No. (%) tested	No. (%) *S. aureus* culture positive	X^2^	*p*-value
Sex				
Male	54	28(51.8)	1.6	0.29
Female	40	26(65)		
Age in years				
< 11	18	9(50)		
11–20	26	19(73.1)	3.87	0.42
21–30	20	11(55)		
31–40	15	8(53.3)		
≥ 41	15	7(46.7)		

### Antimicrobial resistance profile of *S. aureus*

**Fig. 1:**
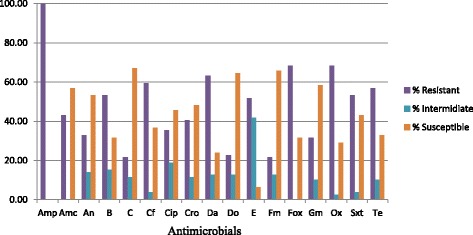
Proportion of susceptible, intermediately resistant and resistant *S. aureus* isolates (*n* = 79) to 17 antimicrobials examined ( Amp = ampicillin; Amc = amoxicillin+clavulanic acid; An = amikacin; B = bacitracin; C = chloramphenicol; Cf = cephalothin; Cip = ciprofloxacin; Cro = ceftriaxone; Fox = cefoxitin; Da = clindamycin Do = doxycycline; E = erythromycin; Gm = gentamicin; Fm = nitrofurantoin; Ox = oxacillin, Sxt = sulfamethoxazole + trimethoprim; Te = tetracycline)

All *S. aureus* isolates examined in the current study were resistant to at least one of the 17 antimicrobials. All (100%) of the isolates were resistant to ampicillin, 54(68.4%) were resistant to oxacillin and cefoxitin, 50(63.3%) to clindanmycin, 47(59.5%) to cephalothin, 45(57%) to tetracycline, 42(53.2%) to sulphamethoxazole+trimethoprim, and bacitracin, 41(51.9%) (Fig. 1).

Based on the sensitivity of isolates to cefoxitin and oxacillin, 54 (68.4%) of the isolates were MRSA. There was no difference in the level of detection of MRSA among *S. aureus* isolates cultured from patients in different age group and from both sexes. Among isolates from various sources, MRSA was detected more frequently in isolates obtained from patients with surgical site infection (*p* = 0.001). Interestingly, all of the 23(100%) of isolates from general surgical and orthopedic wards were MRSA whereas only 31(54.4%) of isolates obtained from patients with ear infection were MRSA (Table 3).

**Table 3:** Relative distribution of MRSA among *S. aureus* isolates from patients of different sex, age group and ward type

Characteristics of source of isolates	No. *S. aureus*	No. MRSA (%)	X^2^	*p* = value
Sex				
Male	42	28(66.7)	0.12	0.87
Female	37	26(70.3)		
Age in years				
< 11	16	12(75)		
11–20	25	16(64)	2.5	0.65
21–30	18	12(66.7)		
31–40	12	7(58.3)		
≥ 41	8	7(87.5)		
Ward type				
General surgical	8	8(100)	15.02	0.001
Orthopedic	15	15(100)		
ENT	56	31(54.4)		

Resistance to two or more antimicrobials was recorded in 74(95%) of the isolates, while resistance to 3 or more antimicrobials was detected in 65(82.3%) of the isolates. Multidrug resistance to 7 or more antimicrobials was detected in 51(64.6%) of the isolates. Majority of isolates cultured from surgical site infection were resistant to several drugs. One of the isolate cultured from a wound swab of patient with surgical site infection from orthopedic ward was found to be resistant to all of the 17 antimicrobials tested (Table 4).

**Table 4:** Resistance pattern of *S. aureus* strains isolated from patients from different wards and specimens

Resistance pattern	No. of drugs to which R is observed	No. (%) isolates	Ward type	Specimen
Amp	1	4(5.1)	ENT	Nasal swab(2)Ear swab(2)
AmpSxt(2),AmpDa,AmpDo, AmpE(2), AmpFox(2), AmpTe(2)	2	10(12.7)	ENT	Ear swab(5), Nasal swab(5)
AmpBSxt, AmpDaOx, AmpTeSxt	3	3(3.8)	ENT	Ear swab(1), Nasal swab(2)
AmpCfFoxOx, AmpDaDoTe,AmpFoxOxSxt,AmpBCfDa	4	4(5.1)	ENT	Ear swab(2) nasal swab(2)
AmpBDoFoxTe, AmpBEOxSxt, AmpDaESxtTe, AmpDoFoxTeOx, AmpBDaETe	5	5(6.3)	ENT(4), Surgical(1)	Ear swab(2), nasal swab(3)
AmpBDaFoxSxtTe, AmpCEFoxOxTe	6	2(2.5)	ENT	Ear swab(1), nasal swab(1)
AmpAnCDaEFoxTe, AmpAnDaFoxOxSxtTe, AmpBCfDaFmFoxOx, AmpBCfCroDaFoxOx, AmpBCfDaFmFoxSxt	7	5(6.3)	ENT(4), Surgical(1)	Ear swab(2), nasal swab(3)
AmpAnCfCroFoxGmOxTe, AmpAnDaDoFoxGmOxTe, AmpBCfDaDoFoxOxTe, AmpBCfDaEOxSxtTe, AmpBCipCroFmGmOxSxt	8	5(6.3)	ENT(2), Surgical(1), Ortho(2),	Nasal swab(1), wound swab(2), ear swab(2),
AmpAnCfDaFoxGmOxSxtTe AmpCCfCipCroDaFoxETe, AmpCCfCipCroFoxGmOx AmpAnBCipDaFoxOxSxtTe, AmpBCCfCroDaFoxGmOx, AmpBCCfCroFoxOxSxtTe	9	6(7.6)	ENT (5), Ortho(1)	Nasal swab(5), ear swab (1)
AmpBCCfCipDaEFmFoxOx, AmpBCCfCipDaEOxSxtTe AmpBCCfCroDaFmFoxOxSxt, AmpBCCfDaDoEFmOxTe	10	4(5.1)	ENT(3), Surgical(1)	Ear swab(3), Wound swab(1)
AmpAnBCfCipDaDoEFoxOxTe, AmpAnBCCfCroDaDoFoxOxTe, AmpAnCCipCroDaEFoxOxSxtTe, AmpAnCCipCroDaEFoxOxSxtTe, AmpAnCCfCipCroDaEFoxGmOx, AmpAmcAnCCfCipEFoxOxSxtTe, AmpAmcBCfCroDaEFmFoxOxSxt, AmpAmcAnBCfCroDaEFmFoxOx, AmpAmcBCfCipCroEFoxOxSxtTe, AmpAnBCfCroDaEFoxGmOxTe	11	9 (11.4)	ENT(6), Ortho(2), Surgical(1)	Ear swab(2), nasal swab(6), wound swab(1)
AmpAnBCfDaDoEFoxGmOxSxtTe, AmpAmcAnBCfDaEFmFoxOxSxtOx, AmpAmcAnBCCfCroDaEFoxOxTe, AmpAmcBCfCipCroEFoxGmOxSxtTe, AmpAmcBCfCipDaDoEFmOxSxtTe	12	5(6.3)	ENT(3), Ortho(2)	Ear swab(2), nasal swab(1), Wound swab(2)
AmpAmcAnCCfCipCroDaEFoxGmOxSxt, AmpAmcBCfCipCroDaEFoxGmOxSxtTe, AmpAmcAnBCfCipCroEFmFoxGmOxSxt, AmpCCfCipCroDaDoEFoxGmOxSxtTe, AmpAnCCfCipCroDaDoFoxGmOxSxtTe, AmpAmcBCCfCipCroDaFoxGmOxSxtTe, AmpAmcBCfCipCroDaFmFoxGmOxSxtTe	13	7(8.9)	ENT(2), Surgical(2), Ortho(3)	Ear swab(2), wound swab(2), nasal swab(3)
AmpAmcAnCCfCipCroDaEFoxGmOxSxtTe, AmpAmcAnBCfCipCroDaEFoxGmOxSxtTe, AmpAmcAnCfCipCroDaDoEFoxGmOxSxtTe, AmpAmcAnBCfCipCroDaEFoxGmOxSxtTe, AmpAmcAnBCCfCipCroDaEFoxOxSxtTe,	14	5(6.3)	Ortho(3), ENT(2)	Nasal swab(4), wound swab(1)
AmpAmcBCCfCipCroDaEFmFoxGmOxSxtTe, AmpAmcAnBCCfCipCroDaEFoxGmOxSxtTe, AmpAmcBCCfCipCroDaDoEFoxGmOxSxtTe	15	3(3.8)	Surgical(1) Ortho(2)	Wound swab (2), nasal swab(1)
AmpAmcAnBCCfCipCroDaDoEFoxGmOxSxtTe	16	1(1.3)	EVNT(1)	Ear swab(1)
AmpAmcAnBCCfCipCroDaDoEFmFoxGmOxSxtTe	17	1(1.3)	Ortho	Wound swab(1)

*Amp* ampicillin, *Amc* amoxicillin+clavulanic acid, *An* amikacin, *B* bacitracin, Cf cephalothin, *C* chloramphenicol, *Cip* ciprofloxacin, *Cro* ceftriaxone, *Fox* cefoxitin, *Da* clindamycin, *Do* doxycycline, *E* erythromycin, *Gm* gentamicin, *Nitro* nitrofurantoin, *Ox* oxacillin, *Sxt* sulfamethoxazole + trimethoprim, *Te* tetracycline, *R* resistant

**Fig. 2:**
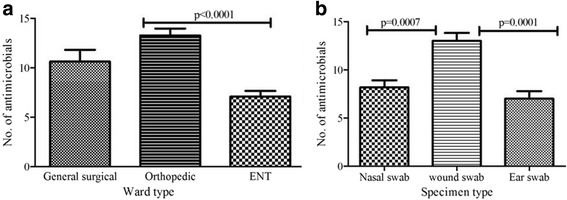
Level of multi-drug resistance among *S. aureus* isolates from different wards and different specimens (Mean number of antimicrobials to which isolates were resistant were compared **a**, Irrespective of specimen type, mean number of drugs to which isolates obtained from orthopedic ward were resistant was significantly higher than those from ENT wards (*p* < 0.0001) **b**, Strains of *S. aureus* isolated from wound swab were MDR to several drugs compared to those obtained from nasal swab (*p* = 0.0007) and ear swab (*p* = 0.0001)

The mean (± SEM) number of drugs to which *S. aureus* isolates obtained from patients from ENT, general surgical ward and orthopedic ward were 7.1 ± 0.6, 10.63 ± 1.2, 13.3 ± 0.7, respectively (Fig. 2a). The level of MDR was significantly higher in *S. aureus* isolates obtained from patients in orthopedic ward compared to those obtained from general surgical ward as well as ENT wards (*p* < 0.0001) while no significant difference was observed among isolates obtained from other two wards. Similarly, comparison of level of MDR among *S. aureus* strains isolated from various specimens revealed that strains isolated from wound swab were resistant to significantly larger number of antimicrobials compared to those isolated from nasal swab as well as ear swab (*p* < 0.001) (Fig. 2b).

## Discussion

The objective of the current study was to assess occurrence and antimicrobial resistance profile of *S. aureus* among patients with surgical site infection and ear infection and corresponding nasal swab at Tikur Anbessa Specialized Hospital. The overall rate of recovery of *S. aureus* among patients with surgical site infection in the current study (47.6–57.1%) is in line with previous studies in which *S. aureus* was shown to be the predominant pathogen responsible for surgical site infection in Ethiopia [6]. Similarly high rate of isolation of *S. aureus* was reported from patients with pus/wound discharge at Gondar University hospital in north Ethiopia [26], and from ear discharges from Hawassa Hospital, southern Ethiopia [27]. In some of the patients with surgical site infection in the current study, *S. aureus* was not detected whereas the same patients were positive from nasal swab. Such heterogeneity could be due to direct topical application of antimicrobials to the infection site which might have affected growth of bacteria from this site. The other possible reason could be the real absence of *S. aureus* from infection site and detection from nasal swab might be due to natural colonization [1]. On the other hand, the absence of *S. aureus* from nasal swab and detection from wound swab and ear discharge could be due to localized infection in the specific infected sites.

Detection of *S. aureus* from ear discharge of 44.1% of patients with otitis media in the current study shows that *S. aureus* is one of the major causes of ear infection in the study population. In previous studies in Ethiopia, *S. aureus* was the dominant bacterial pathogen isolated from patients with otitis media in Mekelle, north Ethiopia [8] and the 2nd predominantly isolated pathogen in northeastern Ethiopia [28]. Unlike previous studies where recovery of bacterial pathogens including *S. aureus* varied among patients with different age group [8, 28], in the current study there was no significant difference in isolation rate of *S. aureus* among age groups, which could be due to small sample size in the current study and due to difference in composition of patient category.

The current study also revealed high rate of resistance to most of the antimicrobials by strains of *S. aureus* isolated from patients with surgical site infection and those with otitis media. Of particular concern is that all strains in this study were resistant to ampicillin unlike previous studies where resistance to ampicillin was observed in 82.2 and 88.5% of *S. aureus* isolates from patients with surgical site infection at Debre Markos Referral Hospital, northwest Ethiopia [29], and patients with otitis media in northeast Ethiopia [28], respectively. On the other hand, it is in agreement with the finding where 100% resistance to ampicillin was recorded in *S. aureus* strains isolated from patients admitted to Felege Hiwot referral Hospital, North Ethiopia [6]. Such difference could be attributed to variation in patient hospital stay, level of infection control practices by health facilities, and previous exposure of patient to antimicrobials [30]. Some isolates from nasal swab and respective wound or ear discharge swab from a single patient had different antimicrobial susceptibility profile. The possible reason for this could be due to localized infection with different strain of *S. aureus* at the wound site while the one from nasal swab could be due to natural colonization.

Rate of occurrence of MRSA (68.4%) out of total *S. aureus* infection in the current study is higher than study reported by Kahsay et al. [29] from patients with surgical site infection at Debre Markos Referral Hospital, northern Ethiopia (49.7%), various clinical specimens from Yekatit 12 Hospital, in Addis Ababa which was 17.5% [24] as well as reports from HIV infected pediatric patients in northwest Ethiopia [31]. It is also higher than a study in Philippines where *S. aureus* isolated from clinical specimens showed 40% MRSA [32]. This could be attributed to difference in composition of patients from whom samples were collected including patients being outpatients, hospitalized, duration of hospital stay and previous antimicrobial use. Majority of MRSA strains in the current study were isolated from hospitalized patients with surgical site infections and those with chronic otitis media who received antimicrobials. Interestingly, all isolates obtained from patients from general surgical and orthopedic wards were MRSA strains, unlike those from ENT ward where only 54% were MRSA suggesting higher rate of infection of patients with MDR strains from the hospital environment in hospitalized patients either during surgical procedure or during post-operative care. Previous study indicated that most of surgical site infections occur during surgery demonstrated by matching of strains of pathogens from surgeon’s fingers and post-operative infection [33].

The probable reason for high rate of overall MDR in *S. aureus* isolates obtained from orthopedic surgery ward compared to those from other wards could be due to high contamination of this specific ward with MDR strains of *S. aureus*. The reason why isolates cultured from nasal swab and ear swab were resistant to less number of drugs compared to those obtained from surgical wound could possibly because the level of previous exposure to antimicrobial agents might not be as high as those from surgical ward who were hospitalized. All isolates from ear discharge and most of the isolates from nasal swab were obtained from outpatients in ENT ward who had less exposure to antimicrobials. MRSA and other MDR nosocomial pathogens can be transmitted through skin contact among patients, from health personnel and hospital environment and surfaces unless proper infection control measures are practiced [34]. The poor sanitary conditions of the hospital and lack of routine surveillance of antimicrobial susceptibility of circulating bacterial strains might have contributed to spread of resistant bacteria in the hospital.

## Conclusion

In general, this study revealed high rate of MDR strains of *S. aureus* in TASH, majority of which being MRSA. As this study involved limited wards in a single hospital and small sample size, it may not represent the whole hospital situation and the country as well. Despite this limitation, the finding warrants implementation of measures to control spread of MDR strains in the hospital and the community. Some of the measures to be undertaken include, improving antimicrobial stewardship through routine monitoring of antimicrobial susceptibility of circulating strains and avoiding or reducing empirical therapy, effective infection control practices, training of health personnel and patients on the risk of antimicrobial resistance.

## Abbreviations

CLSI: Clinical and Laboratory Standards Institute
ENT: Ear nose and throat
MDR: multidrug resistance
MHA: Mueller Hinton agar
MRSA: Methicillin resistant *Staphylococcus aureus*
TASH: Tikur Anbessa Specialized Hospital
TSB: Trypton Soya Broth
